# Quality of End-of-Life Care in Gastrointestinal Cancers: A 13-Year Population-Based Retrospective Analysis in Ontario, Canada

**DOI:** 10.3390/curroncol29120717

**Published:** 2022-11-24

**Authors:** Caitlin SR Lees, Hsien Seow, Kelvin KW Chan, Anastasia Gayowsky, Aynharan Sinnarajah

**Affiliations:** 1 Division of Palliative Medicine, Dalhousie University, Halifax, NS B3H 2Y9, Canada; 2 Department of Oncology, McMaster University, Hamilton, ON L8S 4L8, Canada; 3 Odette Cancer Centre, Sunnybrook Health Sciences Centre, Toronto, ON M4N 3M5, Canada; 4 Institute for Clinical Evaluative Sciences, Master University, Hamilton, ON L8S 4L8, Canada; 5 Division of Palliative Medicine, Queen’s University, Kingston, ON L1G 2B9, Canada

**Keywords:** gastrointestinal cancer, end of life, palliative care, quality indicators, aggressive care, hospital death

## Abstract

Population-based quality indicators of either aggressive or supportive care at end of life (EOL), especially when specific to a cancer type, help to inform quality improvement efforts. This is a population-based, retrospective cohort study of gastrointestinal (GI) cancer decedents in Ontario from 1 January 2006–31 December 2018, using administrative data. Quality indices included hospitalizations, emergency department (ED) use, intensive care unit admissions, receipt of chemotherapy, physician house call, and palliative home care in the last 14–30 days of life. Previously defined aggregate measures of both aggressive and supportive care at end of life were also used. In our population of 69,983 patients who died of a GI malignancy during the study period, the odds of experiencing aggressive care at EOL remained stable, while the odds of experiencing supportive care at EOL increased. Most of our population received palliative care in the last year of life (*n* = 65,076, 93.0%) and a palliative care home care service in the last 30 days of life (*n* = 45,327, 70.0%). A significant number of patients also experienced death in an acute care hospital bed (*n* = 28,721, 41.0%) or had a new hospitalisation in the last 30 days of life (*n* = 33,283, 51.4%). The majority of patients received palliative care in the last year of life, and a majority received a palliative care home service within the last 30 days of life. The odds of receiving supportive care at EOL have increased over time. Differences in care exist according to income, age, and rurality.

## 1. Introduction

Gastrointestinal (GI) malignancies, including esophageal, stomach, liver, pancreatic, colorectal, anus and anal canal, biliary duct, and gallbladder cancers account for a large portion of cancer-related deaths: colorectal cancer alone projected to account for 11% of all cancer deaths in 2021, second only to lung cancer in Canada [1]. In such cases where cancer is advanced and incurable, and a palliative trajectory is clear, appropriate care ensures that patients do not suffer needlessly aggressive care in the last days of life, and instead receive supportive care for symptom management [2].

Quality indicators of whether such patients receive aggressive care or supportive care, and factors associated with the receipt of either, allow policy makers and healthcare providers to understand the quality and cost-effectiveness of care provided and where improvements may be made [2,3,4]. A systematic review of interventions to reduce aggressive care at EOL in patients with cancer found that evidence was significantly limited by the absence of standardised measures [5]. However, a 2020 systematic review of quality indicators for end-of-life (EOL) cancer care identified 15 recommended indicators for “comprehensive and meaningful assessments of quality” that were found to be “scientifically sound” and adequately tested [3]. Many of these indicators are well-suited to study at the population level with administrative data and reflect high-intensity and high-cost acute care that is potentially inappropriate and overly aggressive in advanced cancer [3,6,7].

While a small number of studies have examined aggressive care at EOL for patients with GI malignancies, these studies have used heterogeneous indicators of aggressive care at EOL, and few have considered indicators of supportive care at EOL [8,9,10,11,12,13,14]. As such, we undertook to conduct a comprehensive study of quality indicators of EOL care for GI malignancy decedents in Ontario, Canada with the objective of examining rates of indicators of supportive and aggressive EOL care, as well as factors associated with receiving such care.

## 2. Materials and Methods

This is a population-based, retrospective cohort study of GI cancer decedents in Ontario from 1 January 2006–31 December 2018, with data sourced from administrative data held by ICES (formerly known as the Institute for Clinical Evaluative Sciences). GI cancer was defined as malignancy of the anus, anal canal, or colorectal cancer; esophagus; gallbladder or biliary tract; liver; pancreas; small intestine; and stomach. Table A1 lists the International Classification of Diseases, Tenth Edition (ICD-10) codes used to identify the study population. Patients with invalid provincial health card numbers, ≤18 years of age at death, no known diagnosis of cancer in the Ontario Cancer Registry or without cancer listed as a cause of death, or with a diagnosis of cancer occurring on the same date of death or after death were excluded from analysis. In addition, patients were excluded if they died within 30 days of cancer diagnosis, as it was felt that these cases could introduce bias into our study examining events in the last 30 days of life.

Cases were identified from the Ontario Cancer Registry (OCR), then linked through unique encrypted health insurance number to other administrative healthcare databases, including the Registered Persons Database (RPD), Postal Code Conversion File (PCCF), Canadian Institute for Health Information’s Discharge Abstract Database (DAD), National Ambulatory Care Reporting System (NACRS), Ontario Health Insurance Plan database (OHIP), Resident Assessment Instrument–Contact Assessment (RAICA), and Homecare Database (HCD).

Sociodemographic variables collected included sex, age at death, neighbourhood income quintile, rurality (defined as residence in community of <10,000), GI malignancy subtype at diagnosis, stage at diagnosis, and year of diagnosis. A modified Deyo-Charlson Comorbidity Index score was calculated from between one year and five years prior to death and excluding the GI malignancy resulting in death, based upon ICD-10 codes from hospital admissions [15,16]. Survival was calculated from the date of diagnosis of the GI malignancy subtype. In cases where the GI cause of death did not match exact GI malignancy diagnosis subtype, survival was calculated from the date of most recent cancer diagnosis.

Quality indicators previously developed by Henson et al. were used in this study [3]. Indicators included:

- ≥1 new hospitalizations in the last 30 days of life
- ≥1 emergency department visits in the last 14 or 30 days of life
- ≥1 new intensive care unit (ICU) admission in the last 30 days of life
- Receipt of chemotherapy in the last 14 days of life
- Physician house call in the last 14 days of life
- Palliative home care nursing or support service in the last 30 days of life amongst those not hospitalized for the entirety of the period.

Individual indicators were combined to create two aggregate indicators, aggressive care and supportive care. Aggressive care was defined as receipt in the last 30 days of life of one or more of:

- ≥2 Emergency Department visits
- ≥2 new hospital admissions
- ≥1 new ICU admission in last 30 days of life.

Supportive care was defined as receipt of one or more of:

- ≥1 physician house call in the last 14 days of life
- ≥1 palliative care home care service in last 30 days of life (any provision of service recorded in the HCD and designated as ‘end-of-life’).

Population characteristics at the time of death were analysed using descriptive statistics for each subtype of GI malignancy. The incidence and rate of each quality indicator was calculated for the overall population, by GI malignancy subtype, and by year of death. The Cochran-Armitage trend test was used to determine whether there were statistically significant changes in rates of quality indicators over time. Multivariable logistic regression models were then used to analyse factors associated with a patient receiving either aggressive or supportive care. Covariates employed in the adjusted model were chosen on the basis of the authors’ clinical experience and prior studies [8,9,11,12]. These included age, survival, modified Deyo-Charlson comorbidity index, GI malignancy subtype, neighbourhood income quintile, residency in an urban versus rural area, and year of death.

## 3. Results

In total, 69,983 patients who died from a GI malignancy were included in the study (Table 1). Overall, the proportion of decedents rose by age category and peaked in the 80+ age category (30.7%). Generally, a greater proportion of patients were male than female (57.8% vs. 42.2%), with the exception of gallbladder and biliary tract malignancies, in which women made up the majority of patients. More decedents resided in lower income quintile neighbourhoods and most patients resided in an urban area. Survival varied widely according to GI malignancy subtype, ranging from a mean of 1.30 years (SD 3.02) for pancreatic cancer, to 3.29 years (SD 4.61) for anus, anal canal, and colorectal cancers.

### 3.1. Quality Indicators

Quality indicator rates for all GI malignancy subtypes and overall are shown in Table 2. A substantial proportion of the population experienced death in an acute care bed, with patients spending a mean of 8.36 days (SD 9.79) and a median of 4 days (IQR 0–14) of the last 30 days of life in hospital. The majority of patients received palliative care services within the last year of life (*n* = 65,076, 93.0%).

Of patients who were not hospitalized for the entirety of the last 30 days of life (*n* = 64,780), rates of new hospitalization ranged across GI malignancy subtypes, from 49.0% (*n* = 12,233) in anus, anal canal, and colorectal cancers to 60.2% (*n* = 2332) in liver cancers. The rate of new ICU admission in the last 30 days of life was 5.4% (*n* = 3508) overall, ranging from 3.3% (*n* = 498) in patients with pancreatic cancer to 6.9% (*n* = 530) in patients with esophageal cancer. The majority of patients received palliative care homecare service in the last 30 days of life, 70.0% (*n* = 45,327) overall, and ranging from 66.0% (*n* = 2557) of patients with liver cancer to 74.6% (*n* = 11,154) in patients with pancreatic cancer.

Of patients who were not hospitalised during the last 14 days of life (*n* = 58,106), approximately a third of patients (34.4%, *n* = 20,003) overall had one or more ED visit. Patients with a liver malignancy had a notably higher rate of emergency department usage (44.9%, *n* = 1569). Just 3.7% (*n* = 2158) of patients overall received chemotherapy in the last 14 days of life. Many patients, 30.8% (*n* = 17,920) overall, received a house call from a physician in the last 14 days of life.

Quality indicator aggregates (Table 3) indicated that 17.0% (*n* = 11,021) overall received aggressive care, and 72.3% (46,853) overall received supportive care. Aggressive care was most commonly seen in patients with liver cancer, with 21.6% (*n* = 838) experiencing one or more indicator. Similarly, supportive care was least commonly seen in liver cancer, with 68.4% (*n* = 2650) experiencing an indicator of supportive care.

### 3.2. Factors Associated with Aggressive and Supportive Care

Results of the multivariable logistic regression model for aggregate quality indicators of aggressive and supportive care are shown in Table 4 and Table 5. Younger age, residency in the three lowest income quintile neighbourhoods, and residency in a rural area were all associated with increased odds of experiencing aggressive care at end of life. All GI malignancy subtypes, when compared to anus, anal canal, and colorectal cancers, were found to be associated with increased odds of experiencing aggressive care at end of life, most notably liver cancers and esophageal cancers. Survival for more than 3 months following diagnosis, residency in a rural area, and esophageal, stomach, gallbladder or biliary tract cancer; and residency in a rural area were all associated with increased odds of receiving supportive care in the last 30 days of life.

**Figure 1:**
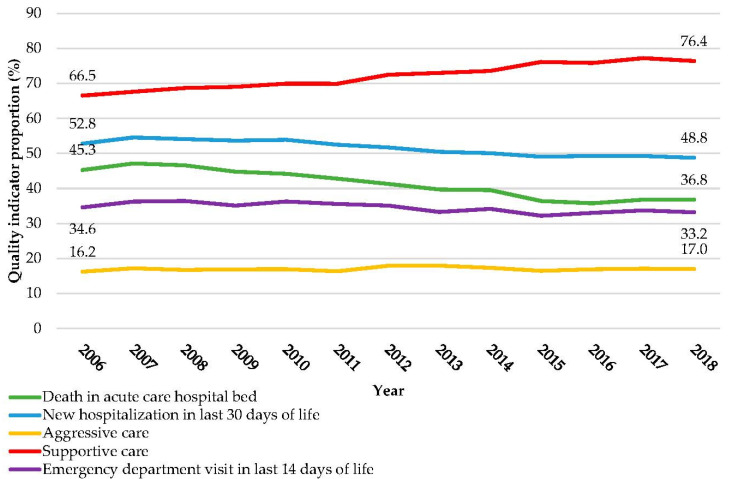
Quality Indicator Proportions by Year of Death.

Trends in individual and aggregate indicators over the 13 years studied are shown in Figure 1. Rates of death in an acute care bed, new hospitalizations in the last 30 days of life, and ED visits in the last 14 days of life all fell significantly over the study period (*p* < 0.0001 for all indicators). The rate of aggressive care remained relatively static over the study period (*p* = 0.4883), while the rate of supportive care increased, from 66.5% (*n* = 2799) in 2006 to 76.4% (*n* = 4387) in 2018 (*p* < 0.0001).

## 4. Discussion

Over the study period, our analysis of Ontario decedents with GI malignancies showed that the odds of experiencing aggressive care at EOL remained stable, while the odds of experiencing supportive care at EOL increased. Most of our population received palliative care services in the last year of life and a palliative care home care service in the last 30 days of life. However, a significant number of patients also experienced death in an acute care hospital bed or had a new hospitalisation in the last 30 days of life. The GI malignancies studied can be difficult to palliate given the breadth of symptoms associated with them and the interventions that may be required for effective palliation [17]. Our large study cohort and the period of time over which outcomes were studied also allow us to consider how the health care utilization and quality of care may have evolved over time.

In our study, we found increased odds of experiencing aggressive care at EOL for patients who were younger, and decreased odds of experiencing aggressive care at EOL for patients with advanced age. These findings are comparable to other studies that have found advanced age is associated with reduced odds of experiencing aggressive care at EOL [8,12,13]. Similarly, advanced age was found to be associated with decreased odds of experiencing supportive care at end of life. While it is tempting to conclude that older patients might have more comorbidities leading to appropriate advance care planning, the comorbidity index was not found to be a significant predictor in our analysis. It is possible that older patients simply decline to engage in more aggressive care measures, rather focussing on more appropriate and home-based care, or that these patients are offered fewer intensive interventions. Interestingly, the association between age and decreased odds of experiencing supportive care at EOL suggests that advanced age results in patients receiving less intensive care more generally, either supportive or aggressive in nature.

Over time, the odds of experiencing aggressive care at EOL remained static, though rates of specific indicators of aggressive care decreased, including an emergency department visit in the last 14 days of life, death in an acute care hospital bed, and new hospitalization in the last 30 days of life. Concurrently, the odds of experiencing supportive care at EOL increased over time, as did rates of specific indicators, suggesting that supportive care interventions either supplant aggressive care measures, alter goals of care, or reduce the need for more aggressive interventions. Given the high symptom burden associated with GI malignancies, our study cohort provides a particularly useful window into how supportive care measures may interact with indicators of aggressive care. Our findings regarding aggressiveness of EOL care are somewhat consistent with previous Ontario based studies [13,18], though direct comparison is hindered by the variable timeframes of the indicators used.

Most of our population received some type of palliative care in the last year of life (*n* = 65,076, 93.0%), and most received a palliative care homecare service in the last 30 days of life (*n* = 45,327, 70.0%). The use of supportive care in the last 30 days of life is comparable to other specific cancers, such as 82.1% in multiple myeloma and 69.8% in gynecologic cancers; though these are historically cancers with lower rates of palliative care utilization at end of life [19,20].

Palliative care is thought to be an important intervention for patients with malignancies, particularly as a part of high-quality end of life care [6,21]. A systematic review found that no one intervention has been found to reduce aggressive EOL care in patients with cancer, it was also noted that evidence has been limited by the absence of standardised indicators of aggressiveness [5], and a few studies have found that palliative care is associated with reduced aggressiveness of care at EOL [9,10,11,12]. Though our study considered indicators of supportive care, the interaction of supportive care interventions with aggressive care interventions was not part of our statistical analysis and may be worthwhile for future study.

Lastly, a significant proportion of our population died in an acute care hospital bed (*n* = 28,721, 41.0%), and a large number also experienced a new hospitalization in the last 30 days of life (*n* = 33,283, 51.4%). These numbers are comparable to other studies of similar populations [8,11,13], and based on previous research, hospitalization close to death and death in an acute care setting is unlikely to align with what patients’ bereaved family members would perceive as ‘excellent’ EOL care [21].

Limitations of our study include the retrospective, observational nature of this study, which leaves us dependent on the documentation of healthcare providers for accurate information in our dataset. Further, the use of administrative data, while useful for capturing data from a large study population, makes it impossible to collect other relevant patient-centred data around EOL care, including preferences for location of death and use of an advance care directive. Despite our large study population, the use of a population located in a single province may limit generalizability of our findings to other regions.

Future studies would benefit from the analysis of the relationship between supportive care and aggressive care interventions, allowing us to better answer the question of whether supportive care interventions are driving the decreased use of aggressive, intensive end of life care. Further, a cost analysis of supportive care interventions would frame the issue from a health economics and policy standpoint.

## 5. Conclusions

Our study is a comprehensive analysis of quality indicators of EOL care for GI malignancy over 13 years in a Canadian province. Findings may serve as a benchmark for future quality improvement initiatives. We found that a large portion of patients are dying in an acute care setting, and hospitalization in the last 30 days of life is common. The odds of experiencing aggressive care at EOL has remained stable over time, with some rates of specific indicators decreasing. The vast majority of patients receive palliative care in the last year of life. The odds of receiving supportive care at EOL have increased over time. Differences in care exist according to income, age, and rurality. Future study of interventions that may improve quality of care, using these benchmarks, are warranted.

## Figures and Tables

**Table 1:** Characteristics of the Study Population.

Indicator		Cancer Subtype															
		Anal and Colorectal		Esophagus		Gallbladder and Biliary		Liver		Pancreas		Small Intestine		Stomach		Total	
		N	%	N	%	N	%	N	%	N	%	N	%	N	%	N	%
Study Population		26,955	100.0	8300	100.0	5683	100.0	4108	100.0	15,964	100.0	949	100.0	8024	100.0	69,983	100.0
Age at death	18–39	281	1.0	48	0.6	67	1.2	42	1.0	82	0.5	13	1.4	129	1.6	662	0.9
	40–49	1014	3.8	288	3.5	197	3.5	136	3.3	511	3.2	72	7.6	459	5.7	2677	3.8
	50–59	2923	10.8	1301	15.7	618	10.9	701	17.1	2130	13.3	123	13.0	1039	12.9	8835	12.6
	60–69	5352	19.9	2311	27.8	1347	23.7	1158	28.2	4047	25.4	221	23.3	1694	21.1	16,130	23.0
	70–79	7398	27.4	2358	28.4	1805	31.8	1261	30.7	4899	30.7	253	26.7	2220	27.7	20,194	28.9
	80+	9987	37.1	1994	24.0	1649	29.0	810	19.7	4295	26.9	267	28.1	2483	30.9	21,485	30.7
Sex	Female	12,453	46.2	1958	23.6	2950	51.9	918	22.3	7790	48.8	442	46.6	3018	37.6	29,529	42.2
	Male	14,502	53.8	6342	76.4	2733	48.1	3190	77.7	8174	51.2	507	53.4	5006	62.4	40,454	57.8
Income Quintile *	1 (lowest)	5590	20.7	1782	21.5	1169	20.6	1069	26.0	3233	20.3	179	18.9	1747	21.8	14,769	21.1
	2	5821	21.6	1814	21.9	1184	20.8	936	22.8	3384	21.2	176	18.5	1741	21.7	15,056	21.5
	3	5241	19.4	1640	19.8	1150	20.2	807	19.6	3122	19.6	208	21.9	1631	20.3	13,799	19.7
	4	5139	19.1	1550	18.7	1069	18.8	645	15.7	3027	19.0	192	20.2	1493	18.6	13,115	18.7
	5 (highest)	5068	18.8	1483	17.9	1095	19.3	628	15.3	3162	19.8	193	20.3	1393	17.4	13,022	18.6
Rural *	Urban	23,300	86.4	6846	82.5	5056	89.0	3693	89.9	13,797	86.4	825	86.9	7229	90.1	60,746	86.8
	Rural	3623	13.4	1444	17.4	620	10.9	409	10.0	2159	13.5	123	13.0	792	9.9	9170	13.1
Comorbidity index	0/missing	12,456	46.2	5642	68.0	3085	54.3	1462	35.6	9982	62.5	450	47.4	5194	64.7	38,271	54.7
	1+	14,499	53.8	2658	32.0	2598	45.7	2646	64.4	5982	37.5	499	52.6	2830	35.3	31,712	45.3
Survival	1–3 mo	3407	12.6	1482	17.9	1510	26.6	1052	25.6	4632	29.0	215	22.7	1772	22.1	14,070	20.1
	3–12 mo	5770	21.4	3680	44.3	2236	39.3	1411	34.3	6756	42.3	290	30.6	3104	38.7	23,247	33.2
	1–5 yr	12,693	47.1	2732	32.9	1633	28.7	1354	33.0	3830	24.0	322	33.9	2644	33.0	25,208	36.0
	5+ yr	5085	18.9	406	4.9	304	5.3	291	7.1	746	4.7	122	12.9	504	6.3	7458	10.7

* Values may not add up to 100% due to missing values.

**Table 2:** Quality indicator rates by GI malignancy subtype.

Indicator	Cancer Subtype															
	Anal and Colorectal		Esophagus		Gallbladder and Biliary		Liver		Pancreas		Small Intestine		Stomach		Total	
	N	%	N	%	N	%	N	%	N	%	N	%	N	%	N	%
**Full Study Population**	**26,955**	**100.0**	**8300**	**100.0**	**5683**	**100.0**	**4108**	**100.0**	**15,964**	**100.0**	**949**	**100.0**	**8024**	**100.0**	**69,983**	**100.0**
Death in acute care hospital bed	10,778	40.0	3700	44.6	2438	42.9	1920	46.7	5950	37.3	426	44.9	3509	43.7	28,721	41.0
Any palliative care service in last year of life	24,328	90.3	7675	92.5	5404	95.1	3861	94.0	15,386	96.4	900	94.8	7522	93.7	65,076	93.0
**Patients not hospitalized for the entirety of last 30 days of life**	**24,940**	**100.0**	**7688**	**100.0**	**5179**	**100.0**	**3875**	**100.0**	**14,961**	**100.0**	**855**	**100.0**	**7282**	**100.0**	**64,780**	**100.0**
New hospitalization in last 30 days of life	12,233	49.0	4264	55.5	2770	53.5	2332	60.2	7343	49.1	455	53.2	3886	53.4	33,283	51.4
New ICU admission in last 30 days of life	1486	6.0	530	6.9	308	5.9	239	6.2	498	3.3	57	6.7	390	5.4	3508	5.4
Any palliative care homecare service in last 30 days of life	16,655	66.8	5530	71.9	3707	71.6	2557	66.0	11,154	74.6	591	69.1	5133	70.5	45,327	70.0
**Patients not hospitalized for the entirety of last 14 days of life**	**22,455**	**100.0**	**6835**	**100.0**	**4572**	**100.0**	**3496**	**100.0**	**13,584**	**100.0**	**763**	**100.0**	**6401**	**100.0**	**58,106**	**100.0**
Emergency department visit in last 14 days of life	7375	32.8	2683	39.3	1493	32.7	1569	44.9	4449	32.8	259	33.9	2175	34.0	20,003	34.4
Chemotherapy use in last 14 days of life	813	3.6	255	3.7	139	3.0	20	0.6	657	4.8	32	4.2	242	3.8	2158	3.7
Physician house call in last 14 days of life	6370	28.4	1983	29.0	1528	33.4	1036	29.6	4796	35.3	233	30.5	1974	30.8	17,920	30.8

**Table 3:** Quality indicator aggregates by GI malignancy subtype.

Indicator	Cancer Subtype															
	Anal and Colorectal		Esophagus		Gallbladder and Biliary		Liver		Pancreas		Small Intestine		Stomach		Total	
	N	%	N	%	N	%	N	%	N	%	N	%	N	%	N	%
**Patients not hospitalized for the entirety of last 30 days of life**	**24,940**	**100.0**	**7688**	**100.0**	**5179**	**100.0**	**3875**	**100.0**	**14,961**	**100.0**	**855**	**100.0**	**7282**	**100.0**	**64,780**	**100.0**
**Aggressive care**	**3695**	**14.8**	**1601**	**20.8**	**869**	**16.8**	**838**	**21.6**	**2562**	**17.1**	**162**	**18.9**	**1294**	**17.8**	**11,021**	**17.0**
At least 2 ED visits in last 30 days of life	3053	12.2	1310	17.0	691	13.3	709	18.3	2149	14.4	129	15.1	1096	15.1	9137	14.1
At least 2 new hospitalizations within last 30 days of life	1702	6.8	747	9.7	430	8.3	418	10.8	1171	7.8	71	8.3	576	7.9	5115	7.9
New ICU admission in last 30 days of life	1486	6.0	530	6.9	308	5.9	239	6.2	498	3.3	57	6.7	390	5.4	3508	5.4
**Supportive care**	**17,255**	**69.2**	**5700**	**74.1**	**3811**	**73.6**	**2650**	**68.4**	**11,551**	**77.2**	**618**	**72.3**	**5268**	**72.3**	**46,853**	**72.3**
Physician house call in last 14 days of life	6389	25.6	1990	25.9	1532	29.6	1041	26.9	4801	32.1	233	27.3	1985	27.3	17,971	27.7
Any palliative care homecare service in last 30 days of life	16,655	66.8	5530	71.9	3707	71.6	2557	66.0	11,154	74.6	591	69.1	5133	70.5	45,327	70.0

**Table 4:** Multivariable Logistic Regression for Aggressive Care.

Indicator		OR	95% CI	
			Lower	Upper
Age at death	18–39	**1.30**	**1.07**	**1.58**
	40–49	0.99	0.89	1.11
	60–69	**0.84**	**0.78**	**0.89**
	70–79	**0.71**	**0.67**	**0.76**
	80+	**0.47**	**0.44**	**0.50**
	50–59 (REF)	1	-	-
Survival	3–12 months	**0.83**	**0.78**	**0.87**
	1–5 years	**0.79**	**0.74**	**0.84**
	5+ years	**0.86**	**0.79**	**0.93**
	1–3 months (REF)	1	-	-
Comorbidity index	1+	1.00	0.96	1.04
	0 or missing (REF)	1	-	-
Cancer subtype	Esophagus	**1.35**	**1.26**	**1.44**
	Gallbladder and biliary	**1.10**	**1.01**	**1.20**
	Liver	**1.43**	**1.31**	**1.55**
	Pancreas	**1.09**	**1.02**	**1.15**
	Small intestine	**1.24**	**1.04**	**1.48**
	Stomach	**1.20**	**1.12**	**1.29**
	Anal and colorectal (REF)	1	-	-
Income quintile	1 (lowest)	**1.12**	**1.05**	**1.20**
	2	**1.10**	**1.03**	**1.17**
	3	**1.11**	**1.04**	**1.19**
	4	1.05	0.98	1.13
	5 (highest) (REF)	1	-	-
Rural status	Rural	**1.91**	**1.81**	**2.02**
	Urban (REF)	1	-	-

Bolded odds ratios indicate statistical significance.

**Table 5:** Multivariable Logistic Regression for Supportive Care.

Indicator		OR	95% CI	
			Lower	Upper
Age at death	18–39	1.13	0.91	1.40
	40–49	**1.23**	**1.09**	**1.39**
	60–69	**0.87**	**0.81**	**0.92**
	70–79	**0.71**	**0.67**	**0.76**
	80+	**0.47**	**0.45**	**0.50**
	50–59 (REF)	1	-	-
Survival	3–12 months	**1.60**	**1.52**	**1.68**
	1–5 years	**1.68**	**1.59**	**1.76**
	5+ years	**1.08**	**1.02**	**1.16**
	0–3 months (REF)	1	-	-
Comorbidity index	1+	**0.93**	**0.90**	**0.96**
	0 or missing (REF)	1	-	-
Cancer subtype	Esophagus	**1.12**	**1.06**	**1.19**
	Gallbladder and biliary	**1.17**	**1.09**	**1.25**
	Liver cancers	**0.88**	**0.81**	**0.95**
	Pancreas	**1.45**	**1.37**	**1.52**
	Small intestine	1.08	0.92	1.26
	Stomach	**1.12**	**1.05**	**1.19**
	Anal and colorectal (REF)	1	-	-
Income quintile	1 (lowest)	**0.69**	**0.65**	**0.73**
	2	**0.84**	**0.79**	**0.89**
	3	**0.91**	**0.86**	**0.97**
	4	**0.94**	**0.88**	**1.00**
	5 (highest) (REF)	1	-	-
Rural status	Rural	**1.23**	**1.16**	**1.30**
	Urban (REF)	1	-	-

Bolded odds ratios indicate statistical significance.

## Data Availability

The data are publicly available in the administrative databases that can be accessed with specific research projects.

## Appendix A

**Table A1:** GI Cancer Cause of Death Codes.

**Cancer Type**	**COD ICD-10 Code**
Esophagus	C150, C151, C152, C153, C154, C155, C158, C159
Stomach	C160, C161, C162, C163, C165, C165, C166, C168, C169
Small Intestine	C170, C171, C172, C173, C178, C179
Colorectal	C180, C181, C182, C183, C184, C185, C186, C187,C188, C189, C19, C199, C200, C209
Anus and anal canal	C210, C211, C212, C218
Liver	C220, C222, C223, C224, C227, C228
Galbladder	C23, C239
Biliary tract	C221, C240, C241, C248, C249
Pancreas	C250, C251, C252, C253, C254, C257, C258, C259
